# Association of *IL-4* Polymorphisms with Allergic Rhinitis in Jordanian Population

**DOI:** 10.3390/medicina56040179

**Published:** 2020-04-14

**Authors:** Baeth Moh’d Al-Rawashdeh, Ahmed Sada Alhanjori, Elnagi Ali, Malek Zihlif

**Affiliations:** 1 Department of Special Surgery, Faculty of Medicine, The University of Jordan, Amman 11942, Jordan; drbraw@gmail.com (B.M.A.-R.); dr.nagihumanity@ju.edu.jo (E.A.); 2 Department of Pharmacology, Faculty of Medicine, The University of Jordan, Amman 11942, Jordan; ahmd.hanjori@gmail.com

**Keywords:** allergic rhinitis, epigenetically, genetically, homozygosity, *IL-4*, multifactorial, polymorphism

## Abstract

*Background and objectives:* Allergic rhinitis has complex patterns of inheritance, and single nucleotide polymorphisms, a common genetic variation in a population, exert a significant role in allergic rhinitis pathology. The current study aimed to investigate the association of *Interleukin-4 (IL-4)* polymorphisms with allergic rhinitis. *Materials and Methods:* Our study included 158 patients with allergic rhinitis and 140 healthy controls from Jordan that were genotyped for *IL-4* single nucleotide polymorphisms (SNPs) C-589T (rs2243250) and T-2979G (rs2227284) using restriction fragment length polymorphism-polymerase chain reaction. Statistical analysis was conducted using IBM SPSS Statistics version 24 software. *Results:* The results showed that the allelic frequency of the minor alleles was 0.19 and 0.67 for C-589T (rs2243250) and T-2979G (rs2227284) in the allergic rhinitis patients, respectively, while it was 0.18 for C-589T (rs2243250) and 0.64 T-2979G (rs2227284) in the control group. The homozygous (TT) genotype of C-589T (rs2243250) was significantly associated with allergic rhinitis (*p* < 0.05), while there was no association of any of T-2979G (rs2227284) genotypes with allergic rhinitis. *Conclusions:* The results of this study indicate that genetic inter-population variation precipitates the differences in the percentages of many diseases among populations, including allergic rhinitis.

## 1. Introduction

Allergic rhinitis (AR) is a common heterogeneous disorder that affects people of all ages. One or more of the following symptoms, including sneezing, itching, nasal congestion, and rhinorrhoea, manifest it. Several factors have been linked to the causing of AR, including pollens, molds, dust mites, and animal dander [1]. Worldwide, AR affects about 10% to 30% of the population, and it is steadily increasing worldwide owing to genetic predisposition, epigenetic events, and environmental exposures. Moreover, sensitization (IgE antibodies) to foreign proteins in the environment exists in up to 40% of the population [2,3]. There is an evident genetic component to the allergic response, which is propelled through mucosal infiltration and effects on plasma cells, mast cells, and eosinophils [1].

Characteristic inflammation of AR is mainly mediated by T-helper cells (TH), which generate different cytokines [4]. TH-1 secretes interleukin-2 (IL-2), interferon-(IFN)γ, and tumor necrosis factor-(TNF)-β, while TH-2 produces IL-4, IL-5, IL-6, and IL-10. Both types of TH cells secrete IL-3, granulocyte-macrophage-colony-stimulating factor (GM-CSF), and TNF-α. IL-4 and IL-13 regulate many cells, including Th2 cells, mast cells, eosinophils, and neutrophils. Both cytokines were found to have a major role in goblet cell hyperplasia, IgE production, adhesion molecules, chemokines, and airway hyperresponsiveness [4]. IL-4 can regulate proliferation, differentiation, and apoptosis in multiple cell types of hematopoietic and non-hematopoietic origin such as myeloid, mast, dendritic, endothelial, muscular, and neuronal cells [5,6,7]. Another T lymphocyte subset, such as Th17 cells, may be involved in the pathogenesis of allergic rhinitis pathogenicity [4]. Th17 cells are known to produce various cytokines, including IL-17, IL-6, TNF-α, and IL-22. Furthermore, anti-IL-17 reduces neutrophil infiltration in an experimental murine asthma model [8,9].

Intriguingly, IL-4 is a pleiotropic protein with diverse biological functions. It is secreted mainly by activated T cells and could reinforce the occurrence and progression of inflammatory reactions characterized by Th2 [4,5,6]. Soluble cytokine, IL-4, has a role in mediating allergic reactions and IgE production. Importantly, mast cells can also secrete IL-4, beside other cytokines such as IL-5 and IL-6 as a consequence of cross-linking of surface IgE, as well as an additional process of allergic inflammation, which augments and amplifies the action of TH-2 cells [10]. Basophils secrete IL-4, too, through a similar activating mechanism as the mast cells and other indirect ones [10]. Cells respond differently to IL-4 depending on the subset to which they belong, which gives rise to different outcomes [11]. IL-4 is the strongest regulatory factor for IgE that plays a significant role in regulating T cells proliferation and differentiation through an acceleration of IgE synthesis. Differentiation imbalance of TH-1/TH-2 leads to abnormal releasing of cytokines, which may have utility in the occurrence and progression of AR [11].

Additionally, IL-4 acts as a cofactor for proliferation and differentiation of B cells, which are inhibited by the antagonized action of (IFN)γ on IL-4. It also switches B cells toward IgE production [10,11,12,13]. In addition, IL-4 promotes proliferation and growth of mast cells, CTL precursors, and thymocytes [12,13,14,15,16,17,18,19,20,21]. Moreover, it has been found that a large amount of IL-4 is produced by activated human ILC2. These newly identified cells were found to be elevated in the tissues of patients with AR, and it was proposed that they might be involved in Th2 priming [22,23,24]. 

In humans, the *IL-4* gene maps on the long arm of chromosome 5 at band q31.1 [24]. A different study showed that *IL-4* promoter and intron-2 polymorphisms (rs2243250; C-589T) and (rs2227284;T2979G), respectively, were associated with AR [25,26]. The overexpression of the *IL-4*, through genetic variation in regulatory regions and its physiological functions, gives it a preeminent role and responsibility for allergic inflammation. Perhaps this IL-4 role is part of a complex relationship between different ILs secreted by TH1 and TH2 and even the newly proposed cells, namely Th17 cells, which are a distinct subpopulation of CD4+ T cells that produce IL-17A, IL-17F, IL-22, TNF-α, and IL-21 [27]. Such a role can demonstrate the distinctive and enduring histological, pathophysiological, and clinical features of allergic inflammation, particularly of AR [10]. 

The current study aimed to investigate the association of *IL-4* polymorphisms with allergic rhinitis. As far as we know, this study is the first to evaluate the associations of *IL-4* polymorphisms and the risk of allergic rhinitis in Jordan. Interestingly this study reported that the TT genotype of C-589T (rs2243250) and not the GG of the T2979G (rs2227284) genotype is significantly associated with allergic rhinitis among Jordanian population. 

## 2. Patients and Methods

This study included 140 controls and 158 patients with AR who attended the University of Jordan Hospital in Amman, Jordan. All patients were consecutively recruited in the current case-control study during the period from July 2018 to July 2019. The controls were recruited from the local community. The inclusion criteria for the controls were having no history of AR diagnosed in the past and having no treatment with antihistamine drugs. The only exclusion criterion for the controls was having a positive skin prick test. AR patients were diagnosed by medical history emphasizing the presence of the following symptoms: sneezing, nasal obstruction, itchy nose, rhinorrhea, clinical examination, and skin prick test (SPT). SPT was conducted using a commercially available SPT kit, which includes various allergens such as cockroach, tree pollen, grass pollen, weeds, mold, dust mite, and animal dander. The patients with positive SPT tests were included in the study group. Results of the skin prick tests were evaluated according to the European Academy of Allergy and Clinical Immunology (EAACI) criteria. Demographic and clinical data were collected by interviewing the participants and referring to their medical records. All participants were of the same ethnicity, and informed consent was signed by all of them. The Ethical Committee of The Scientific Research Deanship ethical committee of The University of Jordan approved the study (reference number of 67/218/1704 on July 4^th^ 2018) [28].

### 2.1. Genotyping of SNPs 

Peripheral blood was collected from all the participants in EDTA-treated tubes. Genomic DNA was extracted using a Wizard® Genomic DNA Purification Kit (Promega Corporation, Wisconsin, USA) and stored at −20 °C. Four microliters of the extracted DNA was diluted with 96 μL of DNA rehydration solution (dilution factor: 25). Absorbance was then measured at a 260 nm wavelength (A260) using an ultraviolet (UV) spectrophotometer to determine DNA concentration (ng/mL), and all samples showed a ratio of more than 1.6.

Genotyping was proceeded for the detection of two SNPs in *IL-4*, C-589T (rs2243250), and T-2979G (rs2227284), using restriction fragment length polymorphism-polymerase chain reaction (RFLP-PCR). PCR was implemented using the MJ Research PTC-100 Thermal Cycler. The primer pairs used for the two SNPs are shown in Table 1.

The PCR reaction was carried out using Go Taq® Green Master Mix (Promega Corporation, USA). 500 ng of extracted DNA and 250 nM of each primer were used. The thermo-cycler profile consisted of 35 cycles. Before the first cycle, initial denaturation cycles of 5 minutes were performed at 95 °C. Each cycle consisted of denaturation at 95 °C for 45 seconds, followed by annealing of primers for 45 seconds at 50 °C for the -589(C/T) SNP and at 58°C for the -2979(T/G) SNP, with primer extension at 72 °C for 45 seconds. Lastly, the temperature was held at 72 °C for 7 minutes to allow the synthesis of non-extended strands. Consequently, gel electrophoresis was used to confirm the existence of the concerned cut DNA fragment.

Restriction fragment length polymorphism was conducted using enzyme digestion for the PCR products of *IL-4* C-589T and T2979G, using their respective restriction enzyme obtained from New England Biolabs (Table 2). The mixture was spun down and incubated at 37 °C for 18 hours. Following this, a preparation of gel electrophoresis was used to perform genotyping of the samples. Next, gel electrophoresis of the digested PCR products was carried out.

The gel was prepared with agarose at a percentage of 3% (3 grams of agarose powder dissolved in 100 mL buffer 1X) and dissolved using microwaves for 1:15 minutes and then 10 µL of ethidium bromide was added. The preparation was poured on its rake with the specified comb. The PCR samples and a 50bp DNA ladder were injected separately into the well. One hundred and four volts was applied for 65 min to facilitate the migration of the DNA fragments regarding their size. Genotypes were performed under UV light by detection of the size of the DNA fragment corresponding to the ladder and the number of existing fragments.

### 2.2. Statistical Analysis

Sociodemographic descriptive data of the study and control groups were analyzed using IBM SPSS Statistics version 24 software. Continuous variables were expressed as mean ± STD and compared using an independent samples t-test, whereas categorical variables were expressed as numbers and percentages and analyzed using the χ^2^ test. Statistical analysis for genotype and allele frequencies between patients and controls was also performed using the IBM SPSS Statistics version 24 software. A *p* value of 0.05 or less was considered to indicate statistical significance for all the data. Odds ratios (ORs) with 95% CIs were used to assess an association between the frequencies of SNPs and case-control status.

## 3. Results

There were 158 patients in the study group and 140 participants in the control group. There was no significant difference between the age means of the two groups (34.9 ± 13.3 vs 35.7 ± 14.6, *p* = 0.615). There were 64 (40.5%) and 55 (39.3%) males in the study and control groups, respectively. There was no significant difference in gender distribution among groups (*P* = 0.771) as shown in Table 3. There were 97 (61.4%) and 88 (62.9%) people with collegial education or above in the study and control groups, respectively. In addition, there were 113 (71.5%) and 102 (72.9%) smokers in the study and control groups, respectively (see Table 3). There were no significant differences regarding the education level and smoking status between groups (*p* = 0.878 and 0.827, respectively).

The prevalence of allergic symptoms among the study group was 139 (88%), 134 (84.8%), 135 (85.4%), and 155 (98.1%) for sneezing, rhinorrhea, nasal blockage, and nasal itching, respectively. The prevalence of various allergens among the study group was 123 (77.9%) for house dust mite, 81 (51.3%) for grass pollen, 119 (75.3%) for tree pollen, 22 (13.9%) for molds, 15 (9.5%) for animal dander (cats and dogs), and 47 (29.7%) for cockroaches (see Table 3). Two *IL-4* SNP genotypes, +589(C/T) SNP and +2979(T/G) SNP, were obtained in patients with AR and controls.

### 3.1. Genotype and Allele Frequencies

It was found that the frequency of homozygosity for the T allele (TT genotype) of C-589T SNP, among the three allelic combinations, is significantly related to AR in Jordanian people (*p* value < 0.05) in comparison with controls. Additionally, it was associated with a 4.17 fold increase in the risk of AR. No significant difference, relative to rs2227284 (T 2979G) SNP, was observed on AR risk between patients and controls in terms of genotype or allele frequencies, as seen in Table 4 and Table 5, respectively.

### 3.2. Haplotype Frequencies

There are no significant additional relationships among haplotypes and AR in Jordanian people. Furthermore, the haplotype TT and GG of rs2243250 and rs2227284, respectively, did not demonstrate any additional increase in the risk of AR between patients with AR and controls (Table 6).

## 4. Discussion

In this study, we explored the association between a single-nucleotide variant in (rs2243250; C-589T) and (rs2227284; T2979G) and AR. The two SNPs were compared with ethnically matched controls. We found a significant association between the TT genotype of C-589T (rs2243250) allergic rhinitis among the Jordanian population. AR is a multifactorial disorder with genetic as well as environmental factors affecting disease development. Additionally, the characterization of AR sensitization has a strong genetic component [3,29]. Undoubtedly, the potency of related allergens, the concentration and duration of exposure, early exposure in infancy or even during fetal life, and viral respiratory infections determine the level of sensitization and the onset of overt AR disease [5].

Exposure to a threshold concentration of allergen excites and activates allergen-specific TH-2 cells in the nasal mucosa of patients with AR leading to an increase in TH-2-type cytokine mRNA expression, specifically IL-4, and eosinophil recruitment in the respiratory passage. The pro-inflammatory process is driven by the released cytokines through the infiltration of the mucosa and actions of mast cells, plasma cells, and eosinophils, which in turn boost the inflammatory response, and the release of cytokines. A cascade of events is stimulated upon repeated exposure to the allergens leading to AR symptoms [1]. These provide undoubted evidence that allergen-activated T cells and their relevant IL-4 production are involved in the development and maintenance of allergic inflammation seen in airway mucosa of patients with allergic respiratory disorders, such as AR [30,31].

IL-4 is a multifunctional cytokine, which plays an important role in immune system regulation. Engagement of the IL-4-to-IL-4 receptor leads to dimerization (or multimerization) of the receptor resulting in a series of phosphorylation events for cellular substrates mediated by receptor-associated kinase and initiation of signaling cascades [9]. Atopic patients with AR have shown a larger number of intra-epithelium dendritic cells than non-allergic patients; in accordance, an intermediation role is played by these cells in the activation of T-cells and, in turn, the release of IL-4. IL-4 plays a key fundamental role in the development of TH-2-like cells, boosting IL-4, IL-5, and the production of other inflammatory cytokines, IgE hyperproduction, and the amplification of TH 2 responses [5]. IL-4 has an effective role in inducing a T-cell to express its gene. Several promoter elements acting together are working to regulate the transcription of the *IL-4* gene. The activation of TH-2-like cells and then lL-4 production ensures the activation of transcription factor synthesis, which is ambidextrous to bind the elements of *IL-4* promoters and to mediate their function [32].

The overexpression of the *IL-4* gene involves an alteration at the molecular level of the gene that preliminarily modulates its regulatory mechanism [5]. Our study of (rs2243250; C-589T) SNP of *IL-4* promoter in Jordanian people reported a significant association of that SNP (TT genotype) with AR susceptibility and was consistent with other studies that emphasized this association in China, Pakistan, and Japan [4,25,33]. However, in contrast, a study of Iranians by Movahedi et al. shows a 7.17 fold increase in the risk of AR with CC genotype of (rs2243250; C-589T) SNP. The T allele in (rs2243250; C-589T) SNP was primarily shown to have enhanced binding to nuclear transcription factors and, in turn, can increase the expression of *IL-4* [34]. Unfortunately, The TC and CC genotypes of rs2243250 (−589 T/C) were linked to an increased gastric cancer susceptibility when compared with the TT genotype in a Chinese population [35]. This discrepancy may be due to variation in allele frequencies in different ethnic groups. For instance, the TT genotype in our population was common, but it was a very rare genotype in the study on the Iranian population. Accordingly, our finding indicates the allergic rhinitis genetic risk factors are heterogeneous between populations. It is known in many other diseases that the genetic risk factors are linked with the occurrence of other SNPs and changes along the DNA, which perhaps is ethnic specific. It is important to point out that the reported association for rs2243250; C-589T should be accepted carefully and requires further validation in the Jordanian population. Such validation can be through the measurement of IL-4 mRNA levels using Real Time quantitative PCR (RTqPCR) upon in vitro activation of lymphocytes in TH2 conditions.

Our study of intron-2 (rs2227284; T2979G) SNP resulted in a non-significant association of this SNP to AR susceptibility in contrast to Michael et al.’s study of Pakistani patients which observed a strong relation between the SNP and AR susceptibility [25]. In addition, we found that studying haplotype frequencies of (rs2243250; C-589T) and (rs2227284; T2979G) SNPs in the IL-4 gene in Jordanian people has no more additional correlation with the susceptibility of AR than (rs2243250; C-589T) SNP alone. 

### Limitations

Our results reflected the patients and controls who are included in the study; although our sample size is considered convenient, increasing the sample size could alter the patient/control ratio of each SNP and provide more accurate results. Furthermore, we could not quantitatively determine a IgE level in both groups (patients and controls) to test for a relation between the SNPs and the elevated level of IL-4 and IgE level.

## 5. Conclusions

The understanding of the genetic background and its correlated polymorphism that is responsible for IL-4 production is obviously of crucial importance in the clarification of the genesis of AR as well as to plan new approaches to prevention and therapy in the future. It was found that C-589T SNP is significantly associated with AR. The results of this study indicate that genetic inter-population variation precipitates the differences in the percentage of many diseases, including AR. Importantly, the result of this study can be added to our previous findings regarding the association between SNPs and allergic airways diseases such as asthma and AR. In three different studies we have reported significant association between many SNPS in ADAM33, GMBS, and FOXO3 genes for asthma and AR. Accordingly, the findings of this study may help in building a clear view for the genetic risk factors of AR among the Jordanian population [36,37,38]. 

## Figures and Tables

**Table 1:** List of primers used for genotyping.

Primers	Primer Sequence	Annealing Temperature	PCR Product
rs2243250_F rs2243250_R	TAAACTTGGGAGAACATGGT	50	195bp
	TGGGGAAAGATAGAGTAATA		
rs2227284_ F rs2227284_R	CTACTCTTGGCAGTTGCTGGAA	58	220bp
	GGAACTCTCTGTAGAATTATGAACTTTAGGTC		

PCR: polymorphism chain reaction, F: forward, R: reverse.

**Table 2:** Genomic sequence polymorphism analysis of *IL-4* gene using RFLP.

rs Number	cDNA Coordinates of SNPs	Position	RE	RFLP Fragments
rs2243250	c.−589 C>T	Promoter	AvaII	C = 177,18, T = 195
rs2227284	c.183+2527T>G	Intron 2	AluI	T = 122,53,45, G = 122,98

cDNA: complementary DNA, SNP: single nucleotide polymorphism, RE: restriction enzyme, RFLP: restriction fragment length polymorphism.

**Table 3:** Demographic and clinical characteristics of the study and control groups.

Demographic and Clinical Categories	Study Group N = 158	Control Group N = 140	Test	*p* value
Age (years) Mean ± STD	34.9 ± 13.3	35.7 ± 14.6	Independent samples t-test	0.615
Male sex N (%)	64 (40.5%)	55 (39.3%)	χ^2^ test	0.771
Collegial education level or above N (%)	97 (61.4%	88 (62.9%)	χ^2^ test	0.878
Smokers N (%)	113 (71.5%)	102 (72.9%)	χ^2^ test	0.827
Nasal symptoms				
Sneezing N (%)	139 (88%)	85 (60.7%)	χ^2^ test	<0.0001
Rhinorrhea N (%)	134 (84.8%)	92 (65.7%)	χ^2^ test	<0.0001
Nasal obstruction N (%)	135 (85.4%)	84 (60%)	χ^2^ test	<0.0001
Nasal itching N (%)	155 (98.1%)	29 (20.7%)	χ^2^ test	<0.0001
SPT results				
House dust mite N (%)	123 (77.9%)	Negative SPT		
Grass pollen N (%)	81 (51.3%)	Negative SPT		
Tree pollen N (%)	119 (75.3%)	Negative SPT		
Molds N (%)	22 (13.9%)	Negative SPT		
Cats and dogs N (%)	15 (9.5%)	Negative SPT		
Cockroaches N (%)	47 (29.7%)	Negative SPT		

SPT: skin prick test.

**Table 4:** Genotype frequencies of *IL-4* polymorphisms in patients with allergic rhinitis (AR) compared to the controls.

		Group		Total	p-Value (z-Score)	Odds Ratio (95% CI)
		Patients	Controls			
rs2243250 (C-589T)	CC	96	88	184	0.71	0.91(0.5727–1.46)
	CT	53	50	103	0.97	1(0.6287–1.62)
	TT	9	2	11	0.05	4.17(0.885–19.63)
rs2227284 (T 2979G)	TT	19	17	36	0.98	0.99(0.5–1.99)
	TG	68	66	134	0.48	0.85(0.536–1.34)
	GG	71	57	128	0.46	1.19(0.75–1.88)

All genotype frequencies are within Hardy-Weinberg equation (χ^2^, *p* value > 0.05).

**Table 5:** Allele frequencies of *IL-4* polymorphisms in patients with allergic rhinitis (AR) compared to the controls.

Alleles	AR Patients	Controls (%)	Total	p-Value (z-Score)	Odds Ratio (95% CI)
rs2243250 (C-589T)					
C	245	226	471	0.34	1.21(0.815–1.8)
T	71	54	125		
rs2227284 (T 2979G)					
T	106	100	206	0.58	1.10(0.785–1.54)
G	210	180	390		

**Table 6:** Frequencies for possible haplotypes in patient group and healthy controls.

rs2243250 (C-589T)			Group		Total	*p* Value (z-Score)	Odds Ratio (95% CI)	
			Patients	Controls				
CC	rs2227284 (T 2979G)	TT	19_a_	17_a_	36	0.94	0.99(0.5–1.99)	
		TG	68_a_	66_a_	134	0.52	0.85(0.54–1.34)	
		GG	9_a_	5_a_	14	0.39	1.63(0.53–4.99)	
	Total		96	88	184			
CT	rs2227284	GG	53_a_	50_a_	103	0.97	1(0.63–1.621)	
	Total		53	50	103			
TT	rs2227284	GG	9_a_	2_a_	11	0.05	4.17(0.89–19.63)	
	Total		9	2	11			
Total	rs2227284 (T 2979G)	TT	19_a_	17_a_	36	0.97	0.99(0.5–1.99)	
		TG	68_a_	66_a_	134	0.48	0.85(0.54–1.34)	
		GG	71_a_	57_a_	128	0.46	1.19(0.75–1.88)	
	Total		158	140	298			

Each subscript letter denotes a subset of group categories whose column proportions do not differ significantly from each other at the 0.05 level. a: 0 cells (0.0%) have expected count less than 5. The minimum expected count is 16.91.
